# Development and validation of the VAE-NT index: a novel biomechanical parameter for distinguishing subclinical corneal abnormalities

**DOI:** 10.3389/fbioe.2025.1598546

**Published:** 2025-07-16

**Authors:** Lanting Yang, Hui Xu, Honghu Jiang, Jingyin Zhu, Shihao Chen

**Affiliations:** ^1^ Department of Ophthalmology, Huadong Hospital, Fudan Universiry, Shanghai, China; ^2^ National Engineering Research Center of Ophthalmology and Optometry, Eye Hospital, Wenzhou Medical University, Wenzhou, China; ^3^ National Clinical Research Center for Ocular Diseases, Eye Hospital, Wenzhou Medical University, Wenzhou, China

**Keywords:** very asymmetric ectasia with normal tomography, dynamic corneal response parameters, technique for order of preference by similarity to ideal solution, keratoconus, corneal biomechanics

## Abstract

**Purpose:**

The aim of this study is to develop an index for distinguishing between very asymmetric ectasia with normal topography (VAE-NT) eyes and normal eyes, with good performance in validity, reliability, and predictive values.

**Methods:**

In the training dataset, this single-center retrospective study involved 102 healthy eyes and 97 VAE-NT eyes. After propensity score matching (PSM), data from 53 healthy eyes and 53 VAE-NT eyes, including demographic and Corvis ST examination results, were collected. The area under the receiver operating characteristic curve (AUC), sensitivity, specificity, intraclass correlation coefficient (ICC), and positive and negative likelihood ratios were calculated for the dynamic corneal response (DCR) parameters of Corvis ST. The Technique for Order of Preference by Similarity to Ideal Solution (TOPSIS) model was used to objectively and comprehensively evaluate the Corvis ST DCRs, and logistic regression was used to determine the optimal combination of parameters that can accurately separate VAE-NT from normal corneas. In the validation dataset, 44 VAE-NT eyes and 49 normal eyes were involved. The validity, reliability, and predictive value of the index were further assessed using the validation dataset. The VAE-NT index was compared with the tomographic and biomechanical index (TBI) in both the training and validation datasets.

**Results:**

In the training dataset, the optimal parameter combination forming the VAE-NT index included the following DCRs: SP A1, SP HC, A1 Time, DA Ratio Max (2 mm), DA Ratio Max (1 mm), Integrated Radius, and stress–strain index version 2 (SSI2). The receiver operating characteristic (ROC) curve analysis showed an AUC value of 0.971, with a cut-off value of 0.425, an accuracy of 95.283%, a specificity of 94.340%, and a sensitivity of 96.230%. In the validation dataset, the AUC value of the VAE-NT index was 0.980. The sensitivity and specificity of the VAE-NT index were 93.180% and 95.920%, respectively. The positive and negative likelihood ratios of the VAE-NT index were 22.830 and 0.071, respectively. The ICC of the VAE-NT index was 0.835, and the accuracy was 94.624%. The VAE-NT index outperformed TBI in both the training and validation datasets.

**Conclusion:**

The VAE-NT index was developed, exhibiting high sensitivity, specificity, and AUC, along with favorable likelihood ratios and repeatability, suggesting that the VAE-NT index is a robust and reliable tool for distinguishing VAE-NT eyes from normal eyes. Further validation in broader populations and over longer follow-up periods is needed to support clinical translation.

## Introduction

Forme fruste keratoconus (FFKC), recently termed very asymmetric ectasia with normal topography (VAE-NT), is a clinically significant condition characterized by normal topography and slit-lamp examination in one eye, whereas the fellow eye shows signs of keratoconus. This atypical manifestation implies an incomplete state of the disease, in which the cornea protrudes, causing irregular astigmatism and vision impairment (Henriquez et al., 2020). Early diagnosis of FFKC is crucial as it enables patients to proactively address the condition and prevent its progression into fully developed keratoconus (KC), thereby mitigating the risk of further vision loss (Rabinowitz, 1998).

The biomechanical properties of KC play a pivotal role in understanding FFKC. KC is a corneal disorder marked by alterations in the normal collagen fibril network, frequently exhibiting asymmetry between the two eyes. It has been proposed that the progression of keratoconus is driven by a biomechanical cycle of decompensation, involving corneal thinning, increased mechanical strain, and stress redistribution, initiated by a localized reduction in corneal material properties (Ruberti et al., 2011; Roberts and Dupps, 2014). In FFKC, subtle biomechanical abnormalities are often present despite the absence of overt clinical findings. These biomechanical alterations may precede morphological changes, potentially leading to progressive corneal protrusion, irregular astigmatism, and visual impairment if left undetected. To improve diagnostic precision, the term VAE-NT has been proposed to replace the previously used designation of FFKC (Ambrósio et al., 2023; Ambrósio et al., 2017; Hwang et al., 2018). Early identification of these subtle biomechanical alterations is critical for the diagnosis of VAE-NT, enabling timely intervention to prevent progression to clinically manifest keratoconus and preserve visual function.

Corneal biomechanics have gained significant attention in recent decades, with their importance recognized in several applications, including the measurement of intraocular pressure, evaluation of ectasia risk following refractive surgeries, and assessment of the effectiveness of corneal cross-linking (CXL) treatment (Roberts and Dupps, 2014; Herber et al., 2021; Ramm et al., 2019; Girard et al., 2015; Piñero and Alcón, 2015). Several *in vivo* methods have been developed to assess corneal biomechanics, among which the Corvis ST (Oculus, Wetzlar, Germany) is widely used. The Corvis ST uses an ultra-fast Scheimpflug camera that captures 140 frames over 32.11 ms, allowing detailed analysis of corneal deformation in response to an air puff stimulus (Tian et al., 2014). Analysis of the resulting deformation yields several dynamic corneal response (DCR) parameters, which correlate with corneal stiffness (Xian et al., 2023; Miao et al., 2023). These metrics include the stiffness parameter at first applanation (SP-A1) (Ambrósio et al., 2017; Zhang et al., 2021a), the deflection amplitude DA, and the ratio between the deflection amplitudes at apex and 2 mm away from apex (DA Ratio Max 2 mm) (Lu et al., 2022). Additional parameters, such as Ambrósio’s relational thickness to the horizontal profile (ARTh) (Tian et al., 2021a), the Corvis biomechanical index (CBI) (Wang et al., 2017), the stress–strain index (SSI) (Zhang et al., 2021b), and the Chinese CBI (cCBI) (Zhang et al., 2024), have all demonstrated clinical utility in the diagnosis of keratoconus (Ren et al., 2021).

The evaluation criteria for diagnostic indicators are primarily based on metrics derived from receiver operating characteristic (ROC) analysis, including the area under the ROC curve (AUC), sensitivity, specificity, and positive and negative likelihood ratios (Mandrekar, 2010). Although an ideal diagnostic indicator should exhibit high performance across all metrics, in practice, some indicators may not simultaneously achieve high AUC, sensitivity, and specificity. Therefore, clinicians often rely on their clinical experience to interpret these metrics, which can further complicate the diagnostic decision-making process. In addition, the intraclass correlation coefficient (ICC) is commonly used to assess the repeatability and stability of diagnostic indicators (Muller R, 1994). Therefore, this study aims to identify diagnostic indicators that demonstrate superior performance in terms of AUC, sensitivity, specificity, and ICC.

Multi-criteria decision analysis (MCDA) is a structured methodology developed to support decision-making processes involving multiple and often conflicting evaluation criteria (Talukder et al., 2018). As described by Keeney, MCDA provides a logical and systematic framework for evaluating options based on multiple criteria (Keeney, 1982). In this study, we used the Technique for Order of Preference by Similarity to Ideal Solution (TOPSIS), which is a widely used MCDA method. Hwang and Yoon (1981) originally proposed that TOPSIS evaluates alternatives based on their geometric proximity to an ideal solution and distance from a negative ideal solution. Due to its computational simplicity and robustness, TOPSIS has become one of the most widely adopted quantitative techniques in multi-criteria decision-making. Its applicability spans various domains and relies on a well-established mathematical foundation. It has been applied for more than three decades (Hwang and Yoon, 1981; Jahanshahloo et al., 2006), with extensive validation and documentation in the scientific literature (Huang et al., 2016; Yoon and Hwang, 1995). In the TOPSIS framework, the optimal alternative is defined as the one with the shortest distance to the positive ideal solution and the greatest distance from the negative ideal solution.

In this study, the validity, reliability, and predictive performance of Corvis ST DCR parameters for identifying VAE-NT were comprehensively evaluated using the TOPSIS approach. Based on sample size requirements and TOPSIS rankings, the seven highest-performing DCR parameters were selected and integrated to construct a novel composite biomechanical index for differentiating VAE-NT from normal corneas.

## Methods

**FIGURE 1:**
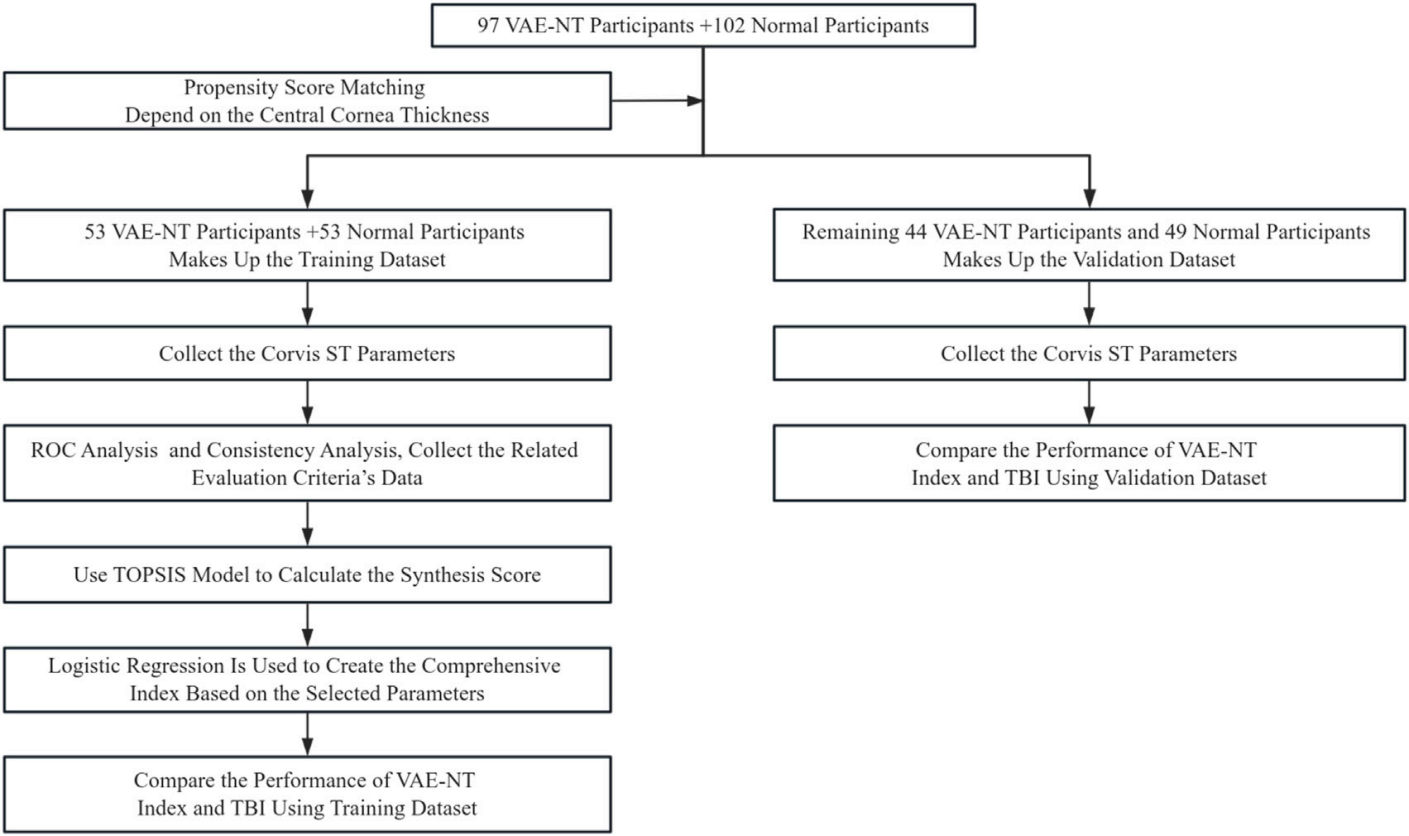
Study workflow and methodology overview.

The steps followed in this study are illustrated in Figure 1.

### Participants

All study participants were recruited at the Eye Hospital of Wenzhou Medical University. This single-center retrospective study initially enrolled 102 healthy eyes and 97 VAE-NT eyes. Each patient underwent a comprehensive eye examination, incorporating tests using the Pentacam and Corvis ST (Oculus Optikgeräte GmbH). The study complied with the ethical principles outlined in the Declaration of Helsinki and was approved by the Ethics Committee of the Eye Hospital of Wenzhou Medical University (ethics approval number: 2022-198-K-154).

In detecting KC, the following criteria were considered: (a) an irregular cornea, determined by distorted keratometry mires and distortion of the retinoscopic, ophthalmoscopic red reflex, or a combination of these and (b) the presence of at least one of the following biomicroscopic signs: Vogt’s striae, Fleischer’s ring of greater than 2-mm arc, or corneal scarring consistent with keratoconus (Rabinowitz, 1998; Arbelaez et al., 2012).

The criteria for very asymmetric ectasia (VAE) refer to the diagnosis of ectasia in one eye, according to previously established definitions, with the fellow eye considered clinically normal based on unremarkable corneal topography; VAE-NT eyes are the fellow eyes of these patients that have normal topography and a keratoconus percentage index (KISA%) score lower than 60, along with a paracentral inferior–superior (I–S value) asymmetry value at 6 mm (3-mm radii) of less than 1.45.

On the other hand, the inclusion criteria for healthy individuals included providing a signed informed consent form, qualification as a candidate for refractive surgery with the absence of topographic distortions (LASIK or SMILE), and having a Corvis ST assessment in the database. The exclusion criteria encompassed any prior ocular surgery or illness, myopia exceeding 10.00 diopters (D), concurrent or prior glaucoma, and the use of hypotonic treatments.

### Corvis ST examinations

All DCR parameters were measured using the same Corvis ST device, and baseline values were recorded. In addition to the biomechanically corrected intraocular pressure (bIOP) and the central corneal thickness (CCT), the Corvis ST provided detailed information on corneal response to an air pulse. To eliminate inter-rater variations, the same technician, blinded to the study design, performed all the examinations. Only results with “OK” in the QS window indicating good image quality were included in the statistical analyses. The Corvis ST provided values of DCRs, including SP A1, DA Ratio Max (2 mm), ARTh, CBI, cCBI, and SSI version 2 (SSI2) (Zhang et al., 2021b). All the information regarding the included Corvis ST parameters is listed in Supplementary Table S1.

### TOPSIS model

We implemented the TOPSIS model to create the VAE-NT index. This work used the TOPSIS method to build an evaluation system (Ramón-Canul et al., 2021).

In this process, positive and negative ideal solutions could be developed using Equation 1 and Equation 2, respectively:

$${\tilde{z}}^{+}=\left({\tilde{z}}_{1}^{+},{\tilde{z}}_{2}^{+},\cdots {\tilde{z}}_{m}^{+}\right)=\left(\max\left\{{\tilde{z}}_{11},{\tilde{z}}_{21},\cdots {\tilde{z}}_{n1}\right\},\max\left\{{\tilde{z}}_{12},{\tilde{z}}_{22},\cdots {\tilde{z}}_{n2}\right\},\cdots ,\max\left\{{\tilde{z}}_{1m},{\tilde{z}}_{2m},\cdots {\tilde{z}}_{nm}\right\}\right),$$ (1)

$${\tilde{z}}^{-}=\left({\tilde{z}}_{1}^{-},{\tilde{z}}_{2}^{-},\cdots {\tilde{z}}_{m}^{-}\right)=\left(\min\left\{{\tilde{z}}_{11},{\tilde{z}}_{21},\cdots {\tilde{z}}_{n1}\right\},\min\left\{{\tilde{z}}_{12},{\tilde{z}}_{22},\cdots {\tilde{z}}_{n2}\right\},\cdots ,\min\left\{{\tilde{z}}_{1m},{\tilde{z}}_{2m},\cdots {\tilde{z}}_{nm}\right\}\right).$$ (2)

The Euclidean distance from the ith $\left(i=1,2,\cdots ,n\right)$ evaluated object to the positive ideal solution is presented in Equation 3:

$$D_{i}^{+}=\sqrt{\sum_{j=1}^{m}W_{j}{\left({\tilde{Z}}_{j}^{+}-{\tilde{z}}_{ij}\right)}^{2}}.$$ (3)

For each evaluation object, its Euclidean distance to the negative ideal solution is presented in Equation 4 as follows:

$$D_{i}^{-}=\sqrt{\sum_{j=1}^{m}W_{j}{\left({\tilde{Z}}_{j}^{-}-{\tilde{z}}_{ij}\right)}^{2}.}$$ (4)

The closeness of the ith $\left(i=1,2,\cdots ,n\right)$ evaluation object to the ideal solution is described in Equation 5:

$$S_{i}=\frac{D_{i}^{-}}{D_{i}^{+}+D_{i}^{-}}.$$ (5)

After normalization, the TOPSIS composite score could be obtained, as shown in Equation 6:

$${S_{i}}^{\prime }=\frac{S_{i}}{\sqrt{\sum_{i=1}^{n}S_{i}}},$$ (6)

where ${S_{i}}^{\prime }$ denotes the proximity of each evaluation object to the optimal solution. 0 ≤ ${S_{i}}^{\prime }$ ≤ 1; the closer ${S_{i}}^{\prime }$ was to 1, the better the evaluation object was.

### Statistical analyses

The Shapiro–Wilk test assessed the normality of continuous variables. Descriptive statistics, including mean ± standard deviation (SD), were used for value description. ROC analysis evaluated the diagnostic efficacy of Corvis ST parameters and the new index for VAE-NT diagnosis. Reliability was assessed using the ICC for averaged measurements, using a two-way random-effects model with “single rater” type and “absolute agreement” ICC definition (Koo and Li, 2016). Propensity score matching (PSM) was used to match CCT between groups, reducing confounding bias. Binary logistic regression with backward stepwise inclusion determined the optimal combination of predictors from individual Corvis ST parameters for creating the VAE-NT index. Due to the requirements for the sample size based on research design and statistical methods, the top seven parameters ranked based on TOPSIS models and AUC values were considered. MedCalc software version 12.3.0.0 (Ostend, Belgium) was used for ROC analysis in both the TOPSIS and AUC groups. R was used for PSM matching. Other statistical analyses were conducted using SPSS (version 22.0, IBM, Inc.). A significance level of 0.05 was applied.

## Results

### Demographic data

Initially, the study included 102 healthy eyes and 97 VAE-NT eyes. After PSM, 53 healthy eyes and 53 VAE-NT eyes were selected for further analysis in the training dataset. The remaining eyes were included in the validation dataset. Table 1a provides information on participants’ age, CCT, bIOP, and gender in the training dataset. The data for CCT in both eyes were comparable (t = −0.019; *P* = 0.850).

**TABLE 1:** (a) Demographic data of the training dataset. (b) Demographic data of the validation dataset.

Parameter	VAE-NT(53 eyes)	Normal (53 eyes)	Test statistic Z	χ^2^	t value	*P*
Age	18.736 ± 5.368	20.264 ± 3.187	−2.077			0.038
Gender	F/M = 11/42	F/M = 16/37		1.242		0.265
CCT	548.148 ± 27.664	549.153 ± 26.904			−0.190	0.850
bIOP	13.947 ± 2.076	16.124 ± 1.672			−5.947	<0.001

Parameter	VAE-NT (44 eyes)	Normal (49 eyes)	Test statistic Z	χ^2^	t value	*P*
Age	20.955 ± 4.779	20.837 ± 3.436	−0.105			0.917
Gender	F/M = 15/29	F/M = 9/40		2.993		0.084
CCT	509.898 ± 16.074	572.553 ± 21.418			15.811	<0.001
bIOP	14.376 ± 1.860	15.894 ± 2.806	−3.117			0.002

Notes: CCT, central cornea thickness; bIOP, biomechanically corrected intraocular pressure.

Table 1b provides information on participants’ age, CCT, bIOP, and gender in the validation dataset. The data for CCT in both eyes were significantly different (t = 15.811; *P* < 0.001).

### Assessment criteria included in the TOPSIS model

The Corvis DCR parameters were assessed in terms of validity, reliability, and predictive values. Validity included AUC, sensitivity, and specificity, while reliability was measured using ICC. The predictive value was evaluated through + LR (positive likelihood ratio) and −LR (negative likelihood ratio). Detailed criteria for evaluation are presented in Table 2.

**TABLE 2:** Assessment criteria included in the TOPSIS model.

Assessment aspect	Assessment indicator	Description of the indicator
Validity	AUC	Area under the receiver operating characteristic curve
	Sensitivity	Proportion of true positive tests out of all patients with a condition
	Specificity	Percentage of true negatives out of all subjects who do not have a disease or condition
Reliability	ICC	Intraclass correlation coefficient is a measure of the correlation between individuals clustered within the same context
Predictive value	+LR	The probability that a positive test would be expected in a patient divided by the probability that a positive test would be expected in a patient without a disease
	−LR	The probability of a patient testing negative who has a disease divided by the probability of a patient testing negative who does not have a disease

### Corvis ST parameters ranked based on the TOPSIS evaluation and AUC scores

After the TOPSIS model analysis, the Corvis ST DCRs were ranked based on the comprehensive evaluation score (Table 3a). Additionally, the ranking according to the AUC score is presented in Table 3b. The results of the ROC analysis and ICC scores for all included Corvis ST parameters are provided in Supplementary Table S2 and Supplementary Table S3, respectively.

**TABLE 3:** (a) Order of Corvis ST parameters based on the TOPSIS model comprehensive evaluation score. (b) Order of Corvis ST parameters based on the AUC score.

Parameter	AUC	Sensitivity	Specificity	+LR	−LR	ICC	US	NS
A1 Time [ms]	0.871	81.130	92.450	10.750	0.200	0.826	0.921	0.062
SP HC	0.701	43.400	96.230	11.500	0.590	0.744	0.780	0.052
SP A1	0.772	62.260	92.450	8.250	0.410	0.602	0.718	0.048
DA Ratio Max (2 mm)	0.660	43.400	94.340	7.670	0.600	0.796	0.670	0.045
CBI	0.710	52.830	92.450	7.000	0.510	0.828	0.667	0.045
Integrated Radius [mm]	0.651	49.060	86.790	3.710	0.590	0.800	0.514	0.034
SSI2	0.733	64.150	77.360	2.830	0.460	0.812	0.502	0.034
DA Ratio Max (1 mm)	0.644	50.940	86.790	3.860	0.570	0.583	0.491	0.033
A1 Velocity [m/s]	0.602	45.280	86.790	3.430	0.630	0.737	0.488	0.033
cCBI	0.674	56.600	79.250	2.730	0.550	0.815	0.487	0.033
HC Time [ms]	0.728	60.380	81.130	3.200	0.490	0.543	0.475	0.032
HC Deflection Area [mm^2^]	0.647	54.720	73.580	2.070	0.620	0.822	0.459	0.031
ARTh	0.591	56.600	69.810	1.870	0.620	0.884	0.458	0.031
Radius [mm]	0.689	52.830	81.130	2.800	0.580	0.612	0.458	0.031
PachySlope [µm]	0.625	64.150	58.490	1.550	0.610	0.893	0.451	0.030
Deformation Amp. Max [mm]	0.651	77.360	47.170	1.460	0.480	0.829	0.450	0.030
HC Deformation Amp. [mm]	0.651	77.360	47.170	1.460	0.480	0.829	0.450	0.030
Peak Dist. [mm]	0.587	92.450	24.530	1.220	0.310	0.783	0.441	0.030
Max Inverse Radius [mm^-1]	0.679	77.360	58.490	1.860	0.390	0.567	0.437	0.029
HC Deflection Length [mm]	0.646	45.280	84.910	3.000	0.640	0.300	0.410	0.027
A1 Deflection Velocity [m/s]	0.517	39.620	79.250	1.910	0.760	0.455	0.379	0.025
A1 Deformation Amp. [mm]	0.559	35.850	86.790	2.710	0.740	0.141	0.368	0.025
A1 Deflection Area [mm^2^]	0.514	30.190	81.130	1.600	0.860	0.472	0.365	0.024
dArc Length Max [mm]	0.614	60.380	67.920	1.880	0.580	0.157	0.361	0.024
Deflection Amp. Max [mm]	0.614	37.740	83.020	2.220	0.750	0.136	0.353	0.024
Deflection Amp Max [ms]	0.543	86.790	28.300	1.210	0.470	0.136	0.351	0.024
A1 dArc Length [mm]	0.534	20.750	92.450	2.750	0.860	0.006	0.349	0.023
HC Deflection Amp. [mm]	0.631	45.280	75.470	1.850	0.730	0.166	0.346	0.023
A1 Deflection Amp. [mm]	0.628	60.380	66.040	1.780	0.600	0.014	0.344	0.023
A1 Deflection Length [mm]	0.590	62.260	60.380	1.570	0.630	0.104	0.339	0.023
HC dArc Length [mm]	0.572	49.060	71.700	1.730	0.710	0.144	0.339	0.023
SSI	0.503	81.130	5.660	0.860	3.330	0.578	0.307	0.021

Parameter	AUC	Sensitivity	Specificity	+LR	−LR	ICC
A1 Time [ms]	0.871	81.130	92.450	10.750	0.200	0.826
SP A1	0.772	62.260	92.450	8.250	0.410	0.602
SSI2	0.733	64.150	77.360	2.830	0.460	0.812
HC Time [ms]	0.728	60.380	81.130	3.200	0.490	0.543
CBI	0.710	52.830	92.450	7.000	0.510	0.828
SP HC	0.701	43.400	96.230	11.500	0.590	0.744
Radius [mm]	0.689	52.830	81.130	2.800	0.580	0.612
Max Inverse Radius [mm^-1]	0.679	77.360	58.490	1.860	0.390	0.567
cCBI	0.674	56.600	79.250	2.730	0.550	0.815
DA Ratio Max (2 mm)	0.660	43.400	94.340	7.670	0.600	0.796
Integrated Radius [mm]	0.651	49.060	86.790	3.710	0.590	0.800
Deformation Amp. Max [mm]	0.651	77.360	47.170	1.460	0.480	0.829
HC Deformation Amp. [mm]	0.651	77.360	47.170	1.460	0.480	0.829
HC Deflection Area [mm^2^]	0.647	54.720	73.580	2.070	0.620	0.822
HC Deflection Length [mm]	0.646	45.280	84.910	3.000	0.640	0.300
DA Ratio Max (1 mm)	0.644	50.940	86.790	3.860	0.570	0.583
HC Deflection Amp. [mm]	0.631	45.280	75.470	1.850	0.730	0.166
A1 Deflection Amp. [mm]	0.628	60.380	66.040	1.780	0.600	0.014
PachySlope [µm]	0.625	64.150	58.490	1.550	0.610	0.893
dArc Length Max [mm]	0.614	60.380	67.920	1.880	0.580	0.157
Deflection Amp. Max [mm]	0.614	37.740	83.020	2.220	0.750	0.136
A1 Velocity [m/s]	0.602	45.280	86.790	3.430	0.630	0.737
ARTh	0.591	56.600	69.810	1.870	0.620	0.884
A1 Deflection Length [mm]	0.590	62.260	60.380	1.570	0.630	0.104
Peak Dist. [mm]	0.587	92.450	24.530	1.220	0.310	0.783
HC dArc Length [mm]	0.572	49.060	71.700	1.730	0.710	0.144
A1 Deformation Amp. [mm]	0.559	35.850	86.790	2.710	0.740	0.141
Deflection Amp Max [ms]	0.543	86.790	28.300	1.210	0.470	0.136
A1 dArc Length [mm]	0.534	20.750	92.450	2.750	0.860	0.006
A1 Deflection Velocity [m/s]	0.517	39.620	79.250	1.910	0.760	0.455
A1 Deflection Area [mm^2^]	0.514	30.190	81.130	1.600	0.860	0.472
SSI	0.503	81.130	5.660	0.860	3.330	0.578

Notes: US, unnormalized score, NS, normalized score.

### VAE-NT index formula

Backward stepwise logistic regression was used to analyze the top seven parameters based on the TOPSIS score, and the following formula was derived:

$$\text{VAE}-\text{NT index}=\mathrm{EXP}\;\left(\text{Beta}\right)/\left(1+\mathrm{EXP}\left(\text{Beta}\right)\right),$$

where Beta = B0+B1 * A1 Time + B2 * SP A1 + B3 *SP HC + B4* DA Ratio Max (2 mm) +B5* DA Ratio Max (1 mm) +B6* SSI2+B7* Integrated Radius.

Moreover, B0 = 117.602, B1 = −19.943, B2 = −0.218, B3 = 1.594, B4 = −5.659, B5 = 52.669, B6 = −11.914, and B7 = −1.427. The results of logistic regression are shown in Table 4.

**TABLE 4:** Variables in the equation based on TOPSIS-selected parameters.

Parameter	β	S.E.	Wald	df	Sig	Exp(*β*)
A1 Time [ms]	−19.943	5.219	14.602	1	<0.001	0.000
SP HC	1.594	0.461	11.982	1	0.001	4.925
SP A1	−0.218	0.060	13.084	1	<0.001	0.804
DA Ratio Max (2 mm)	−5.659	2.761	4.201	1	0.040	0.003
DA Ratio Max (1 mm)	52.669	21.004	6.288	1	0.012	7.477E+22
SSI2	−11.914	5.873	4.115	1	0.043	0.000
Integrated Radius [mm]	−1.427	0.733	3.787	1	0.052	0.240
Constant	117.602	32.822	12.838	1	<0.001	1.185E+51

The regression results of the top seven parameters are based on the AUC value, and the final equation only contains A1 Time, SP HC, SP A1, and HC Time. The details are shown in Supplementary Table S4.

### Assessment of the VAE-NT detection index

**FIGURE 2:**
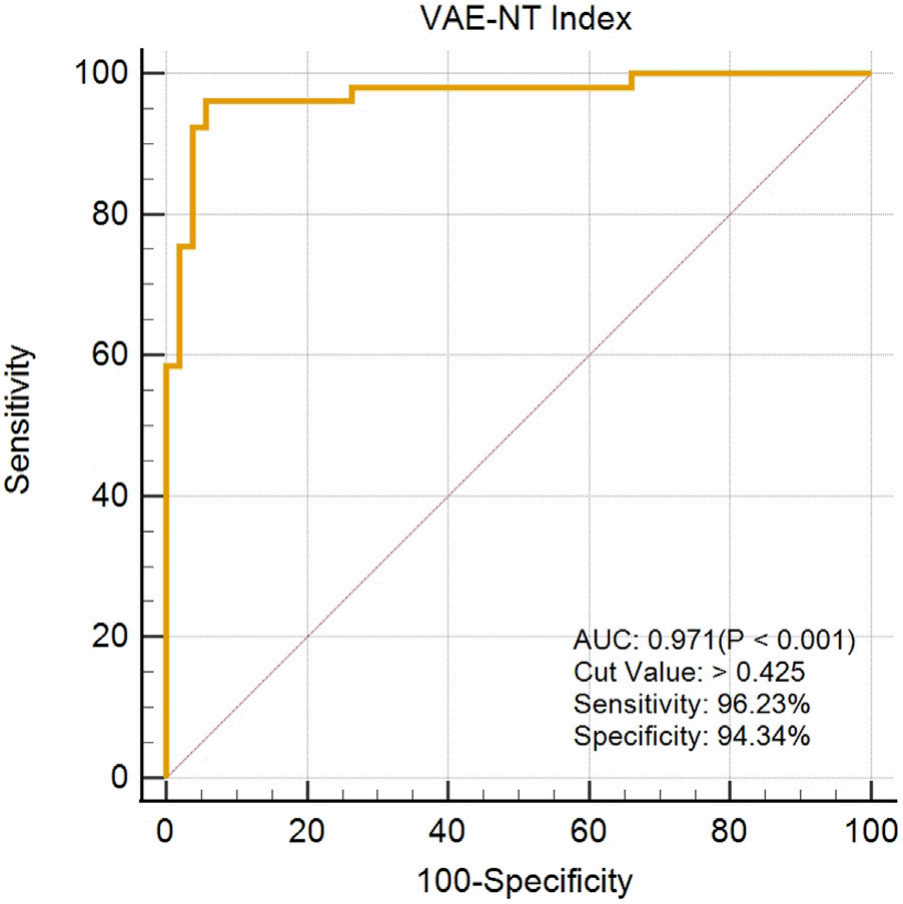
ROC curve of VAE-NT in the training dataset.

**FIGURE 3:**
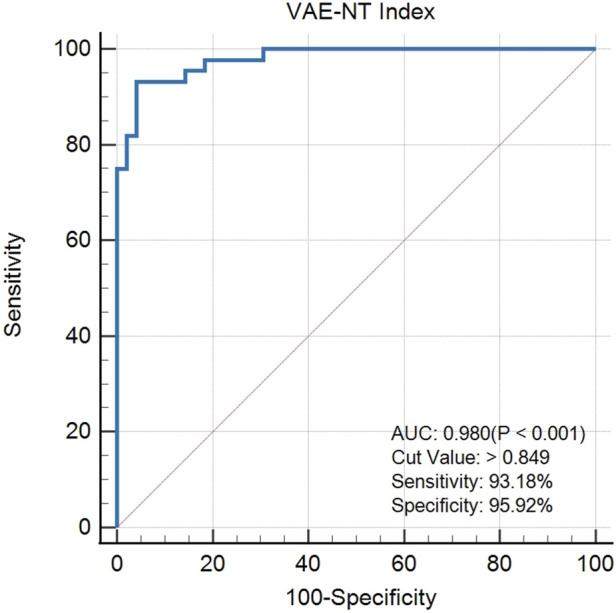
ROC curve of VAE-NT in the validation dataset.

**FIGURE 4:**
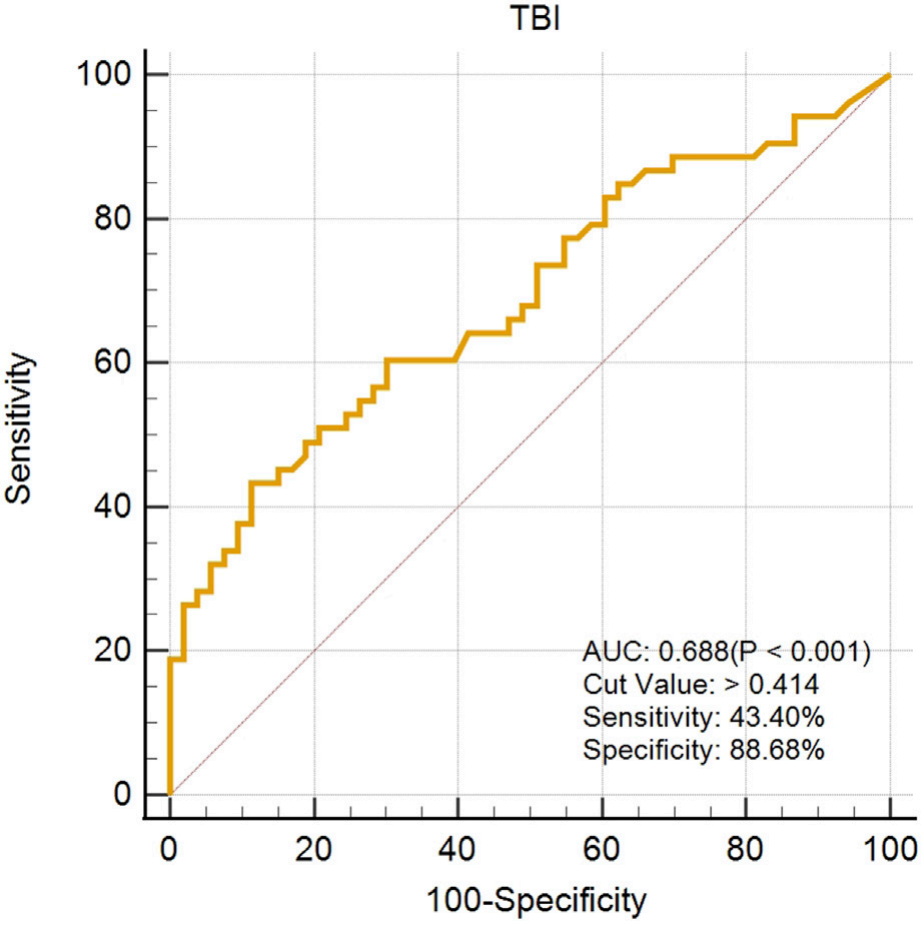
ROC curve of TBI in the training dataset.

**FIGURE 5:**
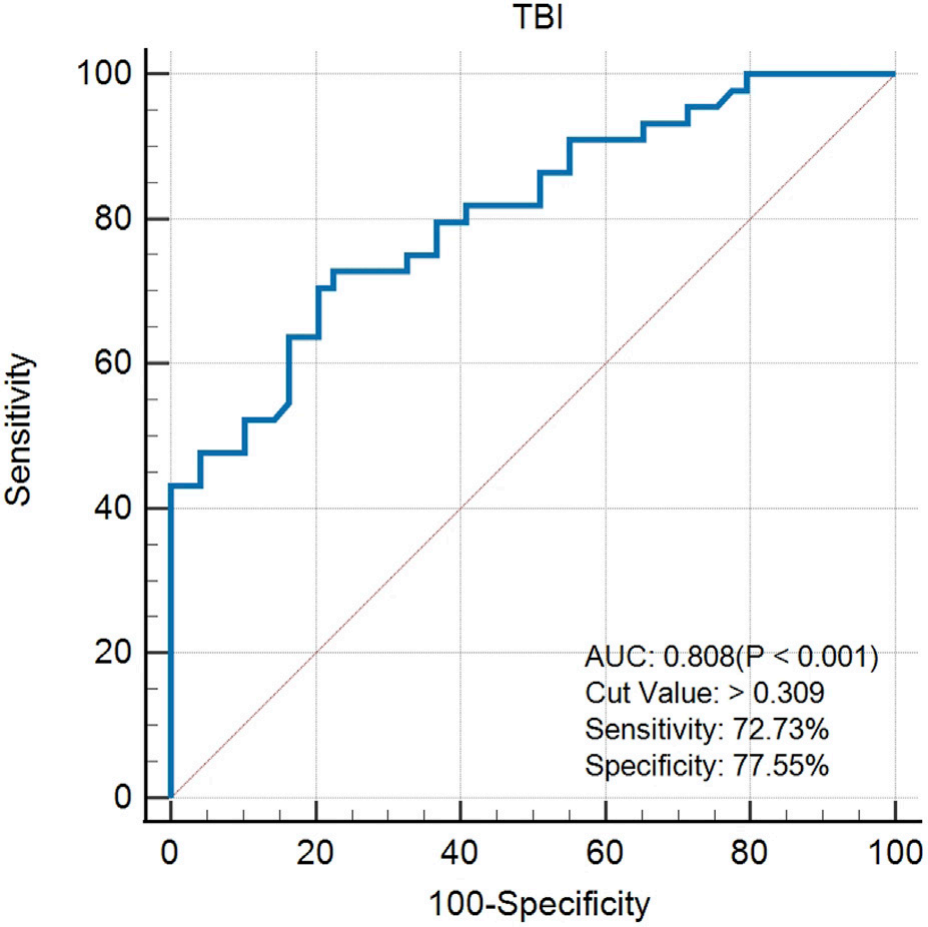
ROC curve of TBI in the validation dataset.

The created VAE-NT index was then tested for validity, reliability, and predictive value in diagnosing VAE-NT from a normal cornea. In the training dataset, the AUC value of the VAE-NT index was 0.971, with a sensitivity of 96.230%, a specificity of 94.340%, and a cutoff value of 0.425. The ICC of VAE-NT was 0.777. Detailed results are presented in Table 5a. Additionally, the AUC value of the composite index based on the AUC value was 0.958, with a sensitivity of 90.570%, a specificity of 92.450%, and a cutoff value of 0.434. Supplementary Table S5 provides detailed results. The AUC value of the tomographic and biomechanical index (TBI) was 0.688, with a sensitivity of 43.400% and a specificity of 88.680%. Detailed results are presented in Table 5b. In the validation dataset, the AUC value of the VAE-NT index was 0.980. The sensitivity and specificity of the VAE-NT index were 93.180% and 95.920%, respectively. The positive and negative likelihood ratios of VAE-NT were 22.830 and 0.071, respectively. The ICC of VAE-NT was 0.835, and the accuracy was 94.624%. Detailed results are presented in Table 5a. For TBI, the receiver operating characteristic curve analysis showed an AUC value of 0.808, with a cutoff value of 0.309, an accuracy of 76.087%, a specificity of 77.550%, and a sensitivity of 72.730%. The positive and negative likelihood ratios were 3.240 and 0.350, respectively. The ICC was 0.881. Detailed results are presented in Table 5b. The ROC curves of VAE-NT and TBI in both the training and validation datasets are shown in Figures 2–5.

**TABLE 5:** (a) Logistic regression results and diagnostic effectiveness evaluation of the VAE-NT index. (b) Diagnostic effectiveness evaluation of TBI.

Dataset	Omnibus test of model coefficients	Hosmer and Lemeshow test	Model accuracy (%)	AUC	Sensitivity (%)	Specificity (%)	+LR	−LR	ICC
Training dataset	<0.001	0.272	95.283	0.971	96.230	94.340	17.000	0.040	0.777
Validation dataset	-	-	94.624	0.980	93.180	95.920	22.830	0.071	0.835

Dataset	Accuracy (%)	AUC	Sensitivity (%)	Specificity (%)	+LR	−LR	ICC
Training dataset	66.038	0.688	43.400	88.680	3.830	0.640	0.751
Validation dataset	76.087	0.808	72.730	77.550	3.240	0.350	0.881

## Discussion

In recent years, several composite diagnostic indices have been developed to diagnose keratoconus. Vinciguerra et al. (2016) introduced the CBI to differentiate between normal and keratoconus corneas. Ambrósio et al. (2017) developed the TBI, which exhibited superior diagnostic performance. Subsequent studies have consistently confirmed their high diagnostic accuracy for keratoconus; however, their performance in detecting FFKC has been comparatively limited (Tian et al., 2021b; Asroui et al., 2022; Wallace et al., 2023; Koh et al., 2019; Ferreira-Mendes et al., 2019; Kataria et al., 2019; Song et al., 2022). Although studies have shown that these indices can achieve an AUC value greater than 0.90, there remains a need to further improve sensitivity, specificity, likelihood ratios (positive and negative), and ICCs (Zhang et al., 2020). This situation requires clinicians to consider multiple diagnostic indices simultaneously when making clinical decisions, thereby increasing the complexity and burden of diagnosis. To address this issue, we developed a novel diagnostic index, VAE-NT. The VAE-NT index demonstrated robust diagnostic performance, achieving high AUC, sensitivity, specificity, favorable likelihood ratios (positive and negative), and ICC values in both the training and validation datasets. Furthermore, compared with the TBI, the VAE-NT index exhibited superior diagnostic efficiency and predictive performance in our dataset.

The primary distinction of the VAE-NT index from previous indices lies in its parameter selection methodology, which is based on the TOPSIS model. Unlike the CBI and TBI, which rely solely on AUC-based parameter selection, the VAE-NT index incorporates a TOPSIS-based multi-criteria evaluation approach (Ambrósio et al., 2017; Vinciguerra et al., 2016). In this study, the TOPSIS model was utilized to evaluate the Corvis ST parameters in terms of validity, predictive power, and reliability. Based on this evaluation, the parameters were ranked according to their TOPSIS-normalized scores. The top seven parameters, determined by the available sample size, were then used in a binary logistic regression analysis, and the model with the optimal diagnostic performance was retained. This study represents a novel integration of the MCDA technique and statistical modeling for the comprehensive evaluation of diagnostic parameters in keratoconus detection.

Corneal thickness is a well-recognized factor influencing the diagnosis of keratoconus. It can significantly affect the diagnostic performance of parameters used to differentiate VAE-NT or KC from normal corneas. It is important to note that corneal thickness was not matched during the development of the CBI and TBI (6, 34). In the present study, the PSM method was applied to balance the distribution of central corneal thickness and other potential confounders between the normal and VAE-NT groups (Kane et al., 2020). The matching process also ensured a consistent sample size across both groups. This approach improves the reliability of the subsequent ROC analysis by meeting sample size requirements and effectively controlling for the potential confounding effects of corneal thickness on diagnostic outcomes. In the validation dataset, which exhibited significant differences in central corneal thickness, the VAE-NT index maintained superior discriminatory ability between VAE-NT and normal eyes. Recently, Ambrósio et al. (2023) introduced the TBI version 2 (TBI_V2_), which showed high diagnostic accuracy for detecting VAE-NT, with an AUC value of 0.945 (DeLong test, P < 0.0001), a sensitivity of 84.4%, and a specificity of 90.1%.

In our dataset, TBI_V2_ demonstrated slightly lower diagnostic performance than the VAE-NT index, although it remains highly effective in identifying VAE-NT. Notably, the VAE-NT index is exclusively based on Corvis ST parameters, which reflect corneal biomechanical properties, whereas TBI_V2_ integrates both biomechanical and tomographic data. These findings highlight the important contribution of biomechanical information to VAE-NT diagnosis, aligning with the well-established role of collagen disruption in keratoconus pathogenesis (Rabinowitz, 1998; Roberts and Dupps, 2014). The components of the VAE-NT index—including SP A1, SSI2, A1 Time, SP HC, DA Ratio Max (2 mm), DA Ratio Max (1 mm), and Integrated Radius—were selected based on their demonstrated effectiveness in distinguishing VAE-NT from normal corneas (Miao et al., 2023; Tian et al., 2021b; Huo et al., 2023; Zhang et al., 2022). Moreover, SP HC, DA Ratio Max (2 mm), DA Ratio Max (1 mm), and Integrated Radius have also been reported to differ significantly between normal and keratoconus corneas (Song et al., 2023).

This study has several limitations. First, the single-center design and relatively moderate sample size (n = 106) may limit the generalizability of the findings. Future studies involving larger, multi-center cohorts are warranted to validate the results. Second, the retrospective nature of data collection may introduce selection bias, highlighting the need for well-designed prospective studies. Third, although the VAE-NT index demonstrated promising diagnostic performance, its validity, reliability, and predictive values require further evaluation using independent external datasets from other clinical centers.

## Conclusion

In this study, the VAE-NT index was developed to distinguish VAE-NT from normal eyes. It demonstrated high sensitivity, specificity, AUC, favorable likelihood ratios, and good reliability, indicating strong diagnostic potential. The use of the TOPSIS model enabled a comprehensive evaluation of diagnostic indicators, facilitating the selection of features with superior overall diagnostic strength and providing clinicians with a more objective decision-making reference. Further validation in larger, more diverse populations and with longer follow-ups is necessary to support clinical implementation.

## Data Availability

The datasets presented in this article are not readily available because the dataset is not publicly available due to ethical/privacy restrictions. Requests to access the datasets should be directed to landy.yang@foxmail.com.

## References

[B1] Ambrósio R. Lopes B. T. Faria-Correia F. Salomão M. Q. Bühren J. Roberts C. J. (2017). Integration of scheimpflug-based corneal tomography and biomechanical assessments for enhancing ectasia detection. J. Refract Surg. 33 (7), 434–443. 10.3928/1081597x-20170426-02 28681902

[B2] Ambrósio R. Machado A. P. Leão E. Lyra J. M. G. Salomão M. Q. Esporcatte L. G. P. (2023). Optimized artificial intelligence for enhanced ectasia detection using scheimpflug-based corneal tomography and biomechanical data. Am. J. Ophthalmol. 251, 126–142. 10.1016/j.ajo.2022.12.016 36549584

[B3] Arbelaez M. C. Versaci F. Vestri G. Barboni P. Savini G. (2012). Use of a support vector machine for keratoconus and subclinical keratoconus detection by topographic and tomographic data. Ophthalmology 119 (11), 2231–2238. 10.1016/j.ophtha.2012.06.005 22892148

[B4] Asroui L. Dagher S. A. Elsheikh A. Lopes B. T. Roberts C. J. Assouad M. (2022). Biomechanical evaluation of topographically and tomographically normal fellow eyes of patients with keratoconus. J. Refract Surg. 38 (5), 318–325. 10.3928/1081597x-20220225-01 35536713

[B5] Ferreira-Mendes J. Lopes B. T. Faria-Correia F. Salomão M. Q. Rodrigues-Barros S. Ambrósio R. (2019). Enhanced ectasia detection using corneal tomography and biomechanics. Am. J. Ophthalmol. 197, 7–16. 10.1016/j.ajo.2018.08.054 30201341

[B6] Girard M. J. A. Dupps W. J. Baskaran M. Scarcelli G. Yun S. H. Quigley H. A. (2015). Translating ocular biomechanics into clinical practice: current state and future prospects. Curr. Eye Res. 40 (1), 1–18. 10.3109/02713683.2014.914543 24832392 PMC4233020

[B7] Henriquez M. A. Hadid M. Izquierdo L. (2020). A systematic review of subclinical keratoconus and forme fruste keratoconus. J. Refract Surg. 36 (4), 270–279. 10.3928/1081597x-20200212-03 32267959

[B8] Herber R. Pillunat L. E. Raiskup F. (2021). Development of a classification system based on corneal biomechanical properties using artificial intelligence predicting keratoconus severity. Eye Vis. 8 (1), 21–11. 10.1186/s40662-021-00244-4 PMC8167942 34059127

[B9] Huang K. W. Huang J. H. Tzeng G. H. (2016). New hybrid multiple attribute decision-making model for improving competence sets: enhancing a company’s core competitiveness. Sustain 8 (2), 175. 10.3390/su8020175

[B10] Huo Y. Chen X. Cao H. Li J. Hou J. Wang Y. (2023). Biomechanical properties analysis of forme fruste keratoconus and subclinical keratoconus. Graefe’s Arch. Clin. Exp. Ophthalmol. 261 (5), 1311–1320. 10.1007/s00417-022-05916-y 36441226

[B11] Hwang C.-L. Yoon K. (1981). “Multiple attribute decision making methods and applications”. New York: Springer-Verlag Berlin Heidelb. 10.1007/978-3-642-48318-9

[B12] Hwang E. S. Perez-Straziota C. E. Kim S. W. Santhiago M. R. Randleman J. B. (2018). Distinguishing highly asymmetric keratoconus eyes using combined scheimpflug and spectral-domain OCT analysis. Ophthalmology 125 (12), 1862–1871. 10.1016/j.ophtha.2018.06.020 30055838 PMC6246819

[B13] Jahanshahloo G. R. Lotfi F. H. Izadikhah M. (2006). Extension of the TOPSIS method for decision-making problems with fuzzy data. Appl. Math. Comput. 181 (2), 1544–1551. 10.1016/j.amc.2006.02.057

[B14] Kane L. T. Fang T. Galetta M. S. Goyal D. K. C. Nicholson K. J. Kepler C. K. (2020). Propensity score matching A statistical method. 33(3):120–122. 10.1097/bsd.0000000000000932 31913173

[B15] Kataria P. Padmanabhan P. Gopalakrishnan A. Padmanaban V. Mahadik S. Ambrósio R. (2019). Accuracy of scheimpflug-derived corneal biomechanical and tomographic indices for detecting subclinical and mild keratectasia in a South Asian population. J. Cataract. Refract Surg. 45 (3), 328–336. 10.1016/j.jcrs.2018.10.030 30527442

[B16] Keeney R. L. (1982). Feature article—decision analysis: an overview. Oper. Res. 30, 803–838. 10.1287/opre.30.5.803 10298708

[B17] Koh S. Ambrósio R. Inoue R. Maeda N. Miki A. Nishida K. (2019). Detection of subclinical corneal ectasia using corneal tomographic and biomechanical assessments in a Japanese population. J. Refract Surg. 35 (6), 383–390. 10.3928/1081597x-20190417-01 31185104

[B18] Koo T. K. Li M. Y. (2016). A guideline of selecting and reporting intraclass correlation coefficients for reliability research. J. Chiropr. Med. 15 (2), 155–163. 10.1016/j.jcm.2016.02.012 27330520 PMC4913118

[B19] Lu N. J. Elsheikh A. Rozema J. J. Hafezi N. Aslanides I. M. Hillen M. (2022). Combining spectral-domain OCT and air-puff tonometry analysis to diagnose keratoconus. J. Refract Surg. 38 (6), 374–380. 10.3928/1081597x-20220414-02 35686708

[B20] Mandrekar J. N. (2010). Receiver operating characteristic curve in diagnostic test assessment. J. Thorac. Oncol. 5 (9), 1315–1316. 10.1097/jto.0b013e3181ec173d 20736804

[B21] Miao Y. Ma X. Qu Z. Eliasy A. Wu B. W. Xu H. (2023). Performance of corvis ST parameters including updated stress-strain index in differentiating between normal, forme-fruste, subclinical, and clinical keratoconic eyes. Am. J. Ophthalmol. 258, 196–207. 10.1016/j.ajo.2023.10.015 37879454

[B46] Muller R. Buttner P. (1994). A critical discussion of intraclass correlation coefficients. Stat. Med. 13, 2465–2476. 10.1002/sim.4780132310 7701147

[B22] Piñero D. P. Alcón N. (2015). Corneal biomechanics: a review. Clin. Exp. Optom. 98 (2), 107–116. 10.1111/cxo.12230 25470213

[B23] Rabinowitz Y. S. (1998). Keratoconus. Surv. Ophthalmol. 42 (4), 297–319. 10.1016/s0039-6257(97)00119-7 9493273

[B24] Ramm L. Herber R. Spoerl E. Raiskup F. Pillunat L. E. Terai N. (2019). Intraocular pressure measurement using ocular response analyzer, dynamic contour tonometer, and scheimpflug analyzer corvis ST. J. Ophthalmol. 2019, 3879651–3879659. 10.1155/2019/3879651 31737355 PMC6815996

[B25] Ramón-Canul L. G. Margarito-Carrizal D. L. Limón-Rivera R. Morales-Carrrera U. A. Rodríguez-Buenfil I. M. Ramírez-Sucre M. O. (2021). Technique for order of preference by similarity to ideal solution (TOPSIS) method for the generation of external preference mapping using rapid sensometric techniques. J. Sci. Food Agric. 101 (8), 3298–3307. 10.1002/jsfa.10959 33222200

[B26] Ren S. Xu L. Fan Q. Gu Y. Yang K. (2021). Accuracy of new corvis ST parameters for detecting subclinical and clinical keratoconus eyes in a Chinese population. Sci. Rep. 11 (1), 4962. 10.1038/s41598-021-84370-y 33654120 PMC7925657

[B27] Roberts C. J. Dupps W. J. Jr (2014). Biomechanics of corneal ectasia and biomechanical treatments. J. Cataract. Refract Surg. 40 (6), 991–998. 10.1016/j.jcrs.2014.04.013 24774009 PMC4850839

[B28] Ruberti J. W. Sinha Roy A. Roberts C. J. (2011). Corneal biomechanics and biomaterials. Annu. Rev. Biomed. Eng. 13, 269–295. 10.1146/annurev-bioeng-070909-105243 21568714

[B29] Song P. Ren S. Liu Y. Li P. Zeng Q. (2022). Detection of subclinical keratoconus using a novel combined tomographic and biomechanical model based on an automated decision tree. Sci. Rep. 12 (0123456789), 5316–5319. 10.1038/s41598-022-09160-6 35351951 PMC8964676

[B30] Song Y. Feng Y. Qu M. Ma Q. Tian H. Li D. (2023). Analysis of the diagnostic accuracy of belin/ambrósio enhanced ectasia and corvis ST parameters for subclinical keratoconus. Int. Ophthalmol. 43 (5), 1465–1475. 10.1007/s10792-022-02543-8 36255612

[B31] Talukder B. Hipel K. W. vanLoon G. W. (2018). Using multi-criteria decision analysis for assessing sustainability of agricultural systems. Sustain Dev. 26 (6), 781–799. 10.1002/sd.1848

[B32] Tian L. Ko M. W. Wang L. K. Zhang J. Y. Li T. J. Huang Y. F. (2014). Assessment of ocular biomechanics using dynamic ultra high-speed Scheimpflug imaging in keratoconic and normal eyes. J. Refract Surg. 30 (11), 785–791. 10.3928/1081597x-20140930-01 25291757

[B33] Tian L. Qin X. Zhang H. Zhang D. Guo L. L. Zhang H. X. (2021b). A potential screening index of corneal biomechanics in healthy subjects, forme fruste keratoconus patients and clinical keratoconus patients. Front. Bioeng. Biotechnol. 9 (9(December), 766605–766610. 10.3389/fbioe.2021.766605 35004638 PMC8733640

[B34] Tian L. Zhang D. Guo L. Qin X. Zhang H. Zhang H. (2021a). Comparisons of corneal biomechanical and tomographic parameters among thin normal cornea, forme fruste keratoconus, and mild keratoconus. Eye Vis. 8 (1), 44–11. 10.1186/s40662-021-00266-y PMC8596950 34784958

[B36] Vinciguerra R. Ambrósio R. Elsheikh A. Roberts C. J. Lopes B. Morenghi E. (2016). Detection of keratoconus with a new biomechanical index. J. Refract Surg. 32 (12), 803–810. 10.3928/1081597x-20160629-01 27930790

[B37] Wallace H. B. Vellara H. R. Gokul A. McGhee C. N. J. Meyer J. J. (2023). Comparison of ectasia detection in early keratoconus using scheimpflug-based corneal tomography and biomechanical assessments. Cornea 42 (12), 1528–1535. 10.1097/ico.0000000000003273 36973879

[B38] Wang Y. M. Chan T. C. Y. Yu M. Jhanji V. (2017). Comparison of corneal dynamic and tomographic analysis in normal, forme fruste keratoconic, and keratoconic eyes. J. Refract Surg. 33 (9), 632–638. 10.3928/1081597x-20170621-09 28880339

[B39] Xian Y. Zhao Y. Sun L. Zhang X. Ding L. Liu Z. (2023). Comparison of bilateral differential characteristics of corneal biomechanics between keratoconus and normal eyes. Front. Bioeng. Biotechnol. 11 (June), 1163223–10. 10.3389/fbioe.2023.1163223 37324412 PMC10267412

[B40] Yoon K. P. Hwang C. L. (1995). Multiple attribute decision making: anintroduction. Berlin Heidelberg: Springer. 10.1007/978-3-642-48318-9

[B41] Zhang H. Eliasy A. Lopes B. Abass A. Vinciguerra R. Vinciguerra P. (2021b). Stress-strain index map: a new way to represent corneal material stiffness. Front. Bioeng. Biotechnol. 9, 640434. 10.3389/fbioe.2021.640434 33777912 PMC7991572

[B42] Zhang H. Tian L. Guo L. Qin X. Zhang D. Li L. (2021a). Comprehensive evaluation of corneas from normal, forme fruste keratoconus and clinical keratoconus patients using morphological and biomechanical properties. Int. Ophthalmol. 41 (4), 1247–1259. 10.1007/s10792-020-01679-9 33389426 PMC8035106

[B43] Zhang H. Zhang X. Hua L. Li L. Tian L. Zhang X. (2022). An exploratory analysis of forme fruste keratoconus sensitivity diagnostic parameters. Int. Ophthalmol. 42 (8), 2473–2481. 10.1007/s10792-022-02246-0 35247116

[B44] Zhang M. Zhang F. Li Y. Song Y. Wang Z. (2020). Early diagnosis of keratoconus in Chinese myopic eyes by combining corvis ST with pentacam. Curr. Eye Res. 45 (2), 118–123. 10.1080/02713683.2019.1658787 31466466

[B45] Zhang P. Yang L. Mao Y. Zhang X. Cheng J. Miao Y. (2024). CorNet: autonomous feature learning in raw corvis ST data for keratoconus diagnosis *via* residual CNN approach. Comput. Biol. Med. 172, 108286. 10.1016/j.compbiomed.2024.108286 38493602

