# Supplementary material for: Development and validation of the VAE-NT index: a novel biomechanical parameter for distinguishing subclinical corneal abnormalities
Source: Front Bioeng Biotechnol. 2025 Jul 16;13:1598546. doi: 10.3389/fbioe.2025.1598546 (PMC12308140; doi:10.3389/fbioe.2025.1598546)
Supplement: Supplementary file 1 [file DataSheet1.zip › Supplementary files/Supplementary table 3.docx]

Supplementary Table 3. Intraclass correlation coefficient of the included Corvis ST parameters

| Parameters | ICC | 95% CI of ICC |
| --- | --- | --- |
| A1 dArc Length [mm] | 0.012 | -0.636 - 0.404 |
| A1 Deflection Amp. [mm] | 0.027 | -0.612 - 0.413 |
| A1 Deflection Area [mm²] | 0.641 | 0.404 - 0.784 |
| A1 Deflection Length [mm] | 0.379 | -0.032 - 0.626 |
| A1 Deflection Velocity [m/s] | 0.626 | 0.378 - 0.775 |
| A1 Deformation Amp. [mm] | 0.248 | -0.253 - 0.548 |
| A1 Time [ms] | 0.905 | 0.842 - 0.943 |
| A1 Velocity [m/s] | 0.848 | 0.748 - 0.909 |
| ARTh | 0.938 | 0.898 - 0.963 |
| CBI | 0.906 | 0.844 - 0.943 |
| cCBI | 0.898 | 0.832 - 0.939 |
| DA Ratio Max (1mm) | 0.736 | 0.562 - 0.841 |
| DA Ratio Max (2mm) | 0.886 | 0.812 - 0.932 |
| dArc Length Max [mm] | 0.272 | -0.189 - 0.557 |
| Deformation Amp. Max [mm] | 0.907 | 0.845 - 0.944 |
| Deflection Amp. Max [mm] | 0.239 | -0.246 - 0.538 |
| Deflection Amp Max [ms] | 0.240 | -0.269 - 0.544 |
| HC dArc Length [mm] | 0.252 | -0.234 - 0.547 |
| HC Deflection Amp. [mm] | 0.285 | -0.176 - 0.567 |
| HC Deflection Area [mm²] | 0.902 | 0.838 - 0.941 |
| HC Deflection Length [mm] | 0.762 | 0.605 - 0.857 |
| HC Deformation Amp. [mm] | 0.907 | 0.845 - 0.944 |
| HC Time [ms] | 0.704 | 0.509 - 0.822 |
| Integrated Radius [mm] | 0.889 | 0.816 - 0.933 |
| Max Inverse Radius [mm^-1] | 0.723 | 0.540 - 0.833 |
| PachySlope [µm] | 0.944 | 0.906 - 0.966 |
| Peak Dist. [mm] | 0.878 | 0.799 - 0.927 |
| Radius [mm] | 0.759 | 0.599 - 0.855 |
| SP A1 | 0.751 | 0.589 - 0.850 |
| SP HC | 0.853 | 0.757 - 0.911 |
| SSI | 0.907 | 0.844 - 0.944 |
| SSI2 | 0.896 | 0.828 - 0.938 |
