# Supplementary material for: Development and validation of the VAE-NT index: a novel biomechanical parameter for distinguishing subclinical corneal abnormalities
Source: Front Bioeng Biotechnol. 2025 Jul 16;13:1598546. doi: 10.3389/fbioe.2025.1598546 (PMC12308140; doi:10.3389/fbioe.2025.1598546)
Supplement: Supplementary file 1 [file DataSheet1.zip › Supplementary files/Supplementary Table 2.docx]

Supplementary Table 2. ROC analysis results of included Corvis ST parameters

| Parameters | ROC | 95% CI of ROC | Youden Index | Sensitivity (%) | Specificity (%) | +LR | -LR |
| --- | --- | --- | --- | --- | --- | --- | --- |
| A1 dArc Length [mm] | 0.534 | 0.434 - 0.631 | 0.132 | 20.750 | 92.450 | 2.750 | 0.860 |
| A1 Deflection Amp. [mm] | 0.628 | 0.529 - 0.720 | 0.264 | 60.380 | 66.040 | 1.780 | 0.600 |
| A1 Deflection Area [mm²] | 0.514 | 0.415 - 0.612 | 0.113 | 30.190 | 81.130 | 1.600 | 0.860 |
| A1 Deflection Length [mm] | 0.590 | 0.491 - 0.685 | 0.226 | 62.260 | 60.380 | 1.570 | 0.630 |
| A1 Deflection Velocity [m/s] | 0.517 | 0.418 - 0.615 | 0.189 | 39.620 | 79.250 | 1.910 | 0.760 |
| A1 Deformation Amp. [mm] | 0.559 | 0.459 - 0.655 | 0.226 | 35.850 | 86.790 | 2.710 | 0.740 |
| A1 Time [ms] | 0.871 | 0.793 - 0.929 | 0.736 | 81.130 | 92.450 | 10.750 | 0.200 |
| A1 Velocity [m/s] | 0.602 | 0.502 - 0.695 | 0.321 | 45.280 | 86.790 | 3.430 | 0.630 |
| ARTh | 0.591 | 0.491 - 0.685 | 0.264 | 56.600 | 69.810 | 1.870 | 0.620 |
| CBI | 0.710 | 0.614 - 0.794 | 0.453 | 52.830 | 92.450 | 7.000 | 0.510 |
| cCBI | 0.674 | 0.576 - 0.762 | 0.359 | 56.600 | 79.250 | 2.730 | 0.550 |
| DA Ratio Max (1mm) | 0.644 | 0.545 - 0.734 | 0.377 | 50.940 | 86.790 | 3.860 | 0.570 |
| DA Ratio Max (2mm) | 0.660 | 0.562 - 0.750 | 0.377 | 43.400 | 94.340 | 7.670 | 0.600 |
| dArc LengthMax [mm] | 0.614 | 0.515 - 0.707 | 0.283 | 60.380 | 67.920 | 1.880 | 0.580 |
| Deformation Amp. Max [mm] | 0.651 | 0.552 - 0.741 | 0.245 | 77.360 | 47.170 | 1.460 | 0.480 |
| Deflection Amp. Max [mm] | 0.614 | 0.514 - 0.707 | 0.208 | 37.740 | 83.020 | 2.220 | 0.750 |
| Deflection Amp Max [ms] | 0.543 | 0.444 - 0.640 | 0.151 | 86.790 | 28.300 | 1.210 | 0.470 |
| HC dArc Length [mm] | 0.572 | 0.472 - 0.668 | 0.208 | 49.060 | 71.700 | 1.730 | 0.710 |
| HC Deflection Amp. [mm] | 0.631 | 0.532 - 0.723 | 0.208 | 45.280 | 75.470 | 1.850 | 0.730 |
| HC Deflection Area [mm²] | 0.647 | 0.549 - 0.738 | 0.283 | 54.720 | 73.580 | 2.070 | 0.620 |
| HC Deflection Length [mm] | 0.646 | 0.547 - 0.737 | 0.302 | 45.280 | 84.910 | 3.000 | 0.640 |
| HC Deformation Amp. [mm] | 0.651 | 0.552 - 0.741 | 0.245 | 77.360 | 47.170 | 1.460 | 0.480 |
| HC Time [ms] | 0.728 | 0.633 - 0.810 | 0.415 | 60.380 | 81.130 | 3.200 | 0.490 |
| Integrated Radius [mm] | 0.651 | 0.552 - 0.741 | 0.359 | 49.060 | 86.790 | 3.710 | 0.590 |
| Max Inverse Radius [mm^-1] | 0.679 | 0.581 - 0.766 | 0.359 | 77.360 | 58.490 | 1.860 | 0.390 |
| PachySlope [µm] | 0.625 | 0.525 - 0.717 | 0.226 | 64.150 | 58.490 | 1.550 | 0.610 |
| Peak Dist. [mm] | 0.587 | 0.487 - 0.682 | 0.170 | 92.450 | 24.530 | 1.220 | 0.310 |
| Radius [mm] | 0.689 | 0.591 - 0.775 | 0.340 | 52.830 | 81.130 | 2.800 | 0.580 |
| SP A1 | 0.772 | 0.680 - 0.848 | 0.547 | 62.260 | 92.450 | 8.250 | 0.410 |
| SP HC | 0.701 | 0.605 - 0.786 | 0.396 | 43.400 | 96.230 | 11.500 | 0.590 |
| SSI | 0.503 | 0.404 - 0.602 | 0.132 | 81.130 | 5.660 | 0.860 | 3.330 |
| SSI2 | 0.733 | 0.638 - 0.814 | 0.415 | 64.150 | 77.360 | 2.830 | 0.460 |
