# Supplementary material for: Development and validation of the VAE-NT index: a novel biomechanical parameter for distinguishing subclinical corneal abnormalities
Source: Front Bioeng Biotechnol. 2025 Jul 16;13:1598546. doi: 10.3389/fbioe.2025.1598546 (PMC12308140; doi:10.3389/fbioe.2025.1598546)
Supplement: Supplementary file 1 [file DataSheet1.zip › Supplementary files/Supplementary table 5.docx]

Supplementary Table 5. Logistic regression results and diagnostic effectiveness evaluation of composite index based on AUC value

|  | Omnibus Tests of  Model Coefficients | Hosmer and  Lemeshow Test | Model Accuracy (%) | AUC | Cut-off | Sensitivity (%) | Specificity (%) | +LR | -LR |
| --- | --- | --- | --- | --- | --- | --- | --- | --- | --- |
| Composite index  based on AUC value | <0.001 | 0.289 | 91.508 | 0.958 | >0.434 | 90.570 | 92.450 | 12.000 | 0.100 |
