# Supplementary material for: Development and validation of the VAE-NT index: a novel biomechanical parameter for distinguishing subclinical corneal abnormalities
Source: Front Bioeng Biotechnol. 2025 Jul 16;13:1598546. doi: 10.3389/fbioe.2025.1598546 (PMC12308140; doi:10.3389/fbioe.2025.1598546)
Supplement: Supplementary file 1 [file DataSheet1.zip › Supplementary files/Supplementary table 4.docx]

Supplementary Table 4. Variables in the Equation based on the parameters selected by AUC

|  | β | S.E. | Wald | df | Sig. | Exp(*β*) |
| --- | --- | --- | --- | --- | --- | --- |
| A1 Time [ms] | -15.989 | 3.679 | 18.892 | 1 | <0.001 | 0.000 |
| SP HC | 1.826 | 0.412 | 19.598 | 1 | <0.001 | 6.207 |
| SP A1 | -0.210 | 0.059 | 12.730 | 1 | <0.001 | 0.811 |
| HC Time [ms] | -1.299 | 0.520 | 6.245 | 1 | 0.012 | 0.273 |
| Constant | 144.670 | 30.872 | 21.959 | 1 | <0.001 | 6.751E+62 |

Notes: A1 Time[ms]: Time from the initiation of the air puff until the first applanation; SP HC: Stiffness parameter at highest concavity; SP A1: Stiffness parameter at applanation 1; HC Time [ms]: Time from the start until highest concavity of the cornea is reached.
